# Light‐intensity physical activity derived from count or activity types is differently associated with adiposity markers

**DOI:** 10.1111/sms.13743

**Published:** 2020-07-05

**Authors:** Charlotte Lund Rasmussen, Melker Staffan Johansson, Patrick Crowley, Peter Fjeldstad Hendriksen, Jørgen Skotte, Nidhi Gupta, Andreas Holtermann

**Affiliations:** ^1^ National Research Center for the Working Environment Copenhagen Denmark; ^2^ Section of Social Medicine Department of Public Health University of Copenhagen Copenhagen Denmark; ^3^ Department of Sports Science and Clinical Biomechanics University of Southern Denmark Odense Denmark

**Keywords:** accelerometer, methodology, physical activity, technical measurements

## Abstract

**Aim:**

The aim of this study was to compare the association between count‐ and activity type–based definitions of light‐intensity physical activity (LIPA) and adiposity markers.

**Methods:**

A total of 516 Danish workers participated in 1‐4 days of hip‐ and thigh‐based accelerometer measurements. Three definitions of average daily time spent in LIPA were derived: *LIPA* (1) time spent between 100 and 2029 CPM, *LIPA* (2) time spent moving and slow walking, and *LIPA* (3) time spent moving, walking slow, and standing. Adiposity markers were body mass index (BMI), body fat percentage, and waist circumference. The cross‐sectional association between the three LIPA definitions and adiposity markers was analyzed and interpreted using compositional regression models followed by reallocation of time between LIPA, moderate‐to‐vigorous physical activity (MVPA), and sedentary behavior (SB), respectively.

**Results:**

The geometric means of daily time (min/day) spent in LIPA 1, LIPA 2, and LIPA 3 were 326, 102, and 274, respectively. We found the direction and strength of the association between the relative importance of daily time spent in LIPA and the adiposity markers to depend on the LIPA definition. For example, reallocating 30 minutes from MVPA to LIPA 1, LIPA 2 and LIPA 3 were associated with a 2.97 (95% CI: 0.68; 5.27), −0.71 (95% CI: −1.43; 0.02), and −0.45 (95% CI: −1.01; 0.11) difference in BMI, respectively.

**Conclusion:**

Our findings highlight the need for caution when comparing results from studies using different definitions of LIPA.

## 1 INTRODUCTION

Most adults spend a substantial proportion of daily waking time in light‐intensity physical activity (LIPA)^1^ which subsequently could have a large impact on health. For example, data from the National Health and Nutrition Examination Survey (NHANES) suggest that adults spend on average 7.8 hours (33%) of a 24‐hour day in LIPA.^2^ In spite of this, the health effects of LIPA are inconclusive. Some studies using count‐based definitions of LIPA have found both positive and negative associations with waist circumference, body fat mass, and body mass index (BMI).^3^, ^4^ In contrast, studies assessing health effects of walking^5^ or standing^6^ have found negative associations with waist circumference and BMI.

One reason for the inconclusive health effects of time spent in LIPA could be the discrepancies in the measurement and definition of LIPA. Although accelerometers are considered to be one of the optimal methods to measure physical behaviors, consensus on how to measure and define LIPA based on accelerometer data is lacking. Consequently, a range of different methods have been used to determine daily time spent in LIPA, with count‐based measurements^3^, ^7^ and activity type–based measurements^6^ being the most common.^8^, ^9^, ^10^

In short, counts express the magnitude of acceleration measured by accelerometers per time unit and can be used to estimate activity intensity and energy expenditure.^8^ This way, counts can be classified into LIPA using cut‐points typically corresponding to pre‐defined thresholds of the metabolic equivalent of tasks (eg, 1.0‐3.0 METS). Another accelerometer‐based method for measuring LIPA is to use both the acceleration and inclination to estimate body postures (eg, sitting and standing) and activity types (eg, walking slow, walking fast, running, and stair climbing). These can then be classified into specific physical activities of low intensity. The two types of measurements often differ in accelerometer placement, being either mounted on the waist or the thigh, respectively.^8^, ^9^, ^10^ Thus, count‐ and activity type–based measurements of LIPA are expressed as daily time spent in either a range of intensities or in specific physical behaviors.

Given that most time awake is spent in low‐intensity activities,^11^ discrepancies in measurements of LIPA could have substantial influence on estimates of daily time spent in LIPA and consequently their association with health. To our knowledge, no study has investigated whether the definition of LIPA affects the associations between daily time spent in LIPA and adiposity markers. Accordingly, the objective of this study was to compare the association between three definitions of LIPA and adiposity markers. LIPA was defined based on the commonly used count‐based definition (ie, 1.0‐3.0 METS), and by grouping physical activities, which could be classified as light intensity based on their MET values (ie, walking slow, moving, and standing). We chose to focus on adiposity markers to facilitate comparability of the current study with previous studies assessing the health effects of LIPA.

## 2 MATERIALS AND METHODS

### 2.1 Study design and study population

This study was based on cross‐sectional accelerometer and questionnaire baseline data from two Danish cohorts: the Danish Physical ACTivity cohort with Objective measurements (DPhacto)^12^, ^13^ and the New Method for Objective Measurements of Physical Activity in Daily Living (NOMAD) study.^14^ The data collection and procedures in the two studies were identical, and thus, the two datasets could be merged.^15^

The study population consisted of workers from Danish companies within transportation, cleaning, manufacturing, construction, road maintenance, garbage disposal, assembly, mobile plant operation, and health services.^12^, ^13^, ^14^ Eligible workers were between 18 and 65 years old and employed for at least 20 hours/week and had given informed consent to participate. Workers were excluded if they were pregnant and had fever on the day of testing or allergy to adhesives.

### 2.2 Data collection

The data collection and procedures have been described in detail previously.^12^, ^13^, ^14^ In short, eligible workers were invited to complete a questionnaire and to participate in a physical health examination, consisting of anthropometric measurements at baseline. Participants were also asked to wear accelerometers for four consecutive days with a minimum of two consecutive workdays and to keep a record of their work hours, time in bed, and periods of non‐wear time in a diary.

### 2.3 Ethical considerations

The DPhacto and NOMAD studies were approved by the Danish data protection agency and local Ethics Committee (file number H‐2‐2012‐011^12^ and file number H‐2‐2011‐047,^14^ respectively). Both studies were conducted according to the Helsinki Declaration, and all data were anonymized in relation to individuals and workplaces.

### 2.4 Eligibility criteria

**FIGURE 1:**
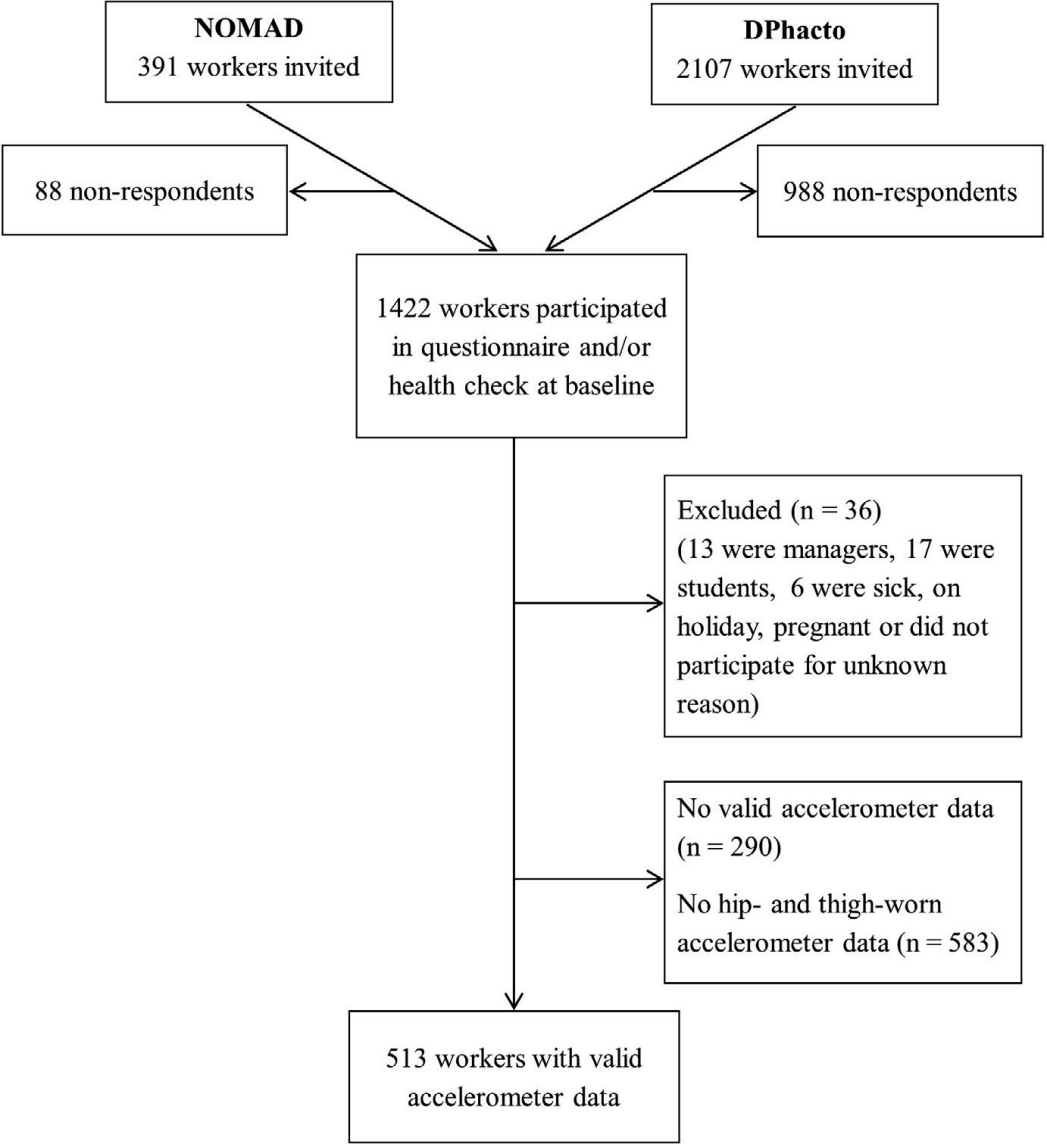
Flowchart of participants in the NOMAD and DPhacto study included in the current paper

Flow of the study participants is shown in Figure 1. A total of 1422 workers participated in the baseline questionnaire and/or health check, of which 36 were excluded because they were managers, students, on holiday, pregnant, or for unknown reasons; 290 did not fulfill the criterion of having one valid day of technical measurements; and 583 did not have both hip‐ and thigh‐worn accelerometer data. Thus, a total of 513 workers were included in the analyses.

One valid day of technical measurements consisted of at least 10 hours of accelerometer measurements during waking hours and at least one measurement of time in bed at night. Moreover, to ensure the same individuals were included in all analyses (ie, regardless of LIPA definition), only individuals with both valid hip‐ and thigh‐worn accelerometer data were included. Participants could be missing hip‐worn accelerometer data, as we changed the procedure for accelerometer from thigh and hip to thigh and lower back during data collection. Comparing the characteristics of individuals included and excluded from the analysis showed no notable difference (see Table S1).

### 2.5 Accelerometer measurements of physical activities and body postures

Daily time spent in physical activity types and body postures (ie, sitting and standing) was assessed using data from two tri‐axial ActiGraph GT3X + accelerometers (ActiGraph). The accelerometers were placed on the right iliac crest and the right frontal thigh using double‐sided adhesive tape (3 M, Hair‐Set) and Fixomull (Fixomull BSN medical GmbH).^16^ Accelerometer data were downloaded using ActiLife software version 5.5.^17^

Daily time use in physical activity and stationary behaviors was based on either counts per minute (CPM) or physical activity–type measurements. For the CPM‐based measurement, data from the hip‐worn accelerometer were used. Sedentary behavior (SB), LIPA, and moderate‐to‐vigorous physical activity (MVPA) were categorized using the Freedson et al cut‐points in the ActiLife software (ActiGraph h).^18^ For the measurements based on physical activity type, data from the thigh‐worn accelerometer were used and analyzed using the Acti4 software (The National Research Centre for the Working Environment, Denmark and The Federal Institute for Occupational Safety and Health, Germany [BAuA]).^19^ The Acti4 software detects physical activity types and body postures (ie, cycling, stair climbing, running, walking, moving, standing, sitting, and/or lying) with high sensitivity and specificity.^19^, ^20^

### 2.6 Outcomes: adiposity markers

The outcome indicators of adiposity were body mass index (BMI), body fat percentage, and waist circumference. Waist circumference was measured at the approximate midpoint between the uppermost lateral border of the iliac crest and the last palpable rib. The measurement was taken at maximal expiration during relaxed normal breathing using a measurement tape (Seca, model 201)^21^ with the average of two measurements recorded. Weight and body fat percentage were measured without shoes and socks using a bio‐impedance segmental body composition analyzer (Tanita model BC418 MA)^22^ to the nearest 0.1%. Height was measured without shoes using a stadiometer (Seca, model 201) to the nearest 0.1 cm. BMI was calculated as body mass (in kg) divided by height (in m) squared (kg/m^2^).

### 2.7 Exposure: daily time‐use compositions

We used three definitions of LIPA: (1) average daily time spent with activity CPM of 100‐2029 (*LIPA 1*); (2) average daily time spent moving and walking slow (*LIPA 2*); and (3) average daily time spent moving, walking slow, and standing (*LIPA 3*). Walking slow was defined as walking with a cadence of ≤100 steps/min.^23^ The definitions of LIPA 1 and LIPA 2 were based on activities, which could be classified as low‐intensity activities based on their estimated MET values (ranging from 1.5 to 3.0).^24^, ^25^

For each of the three LIPA definitions, the following compositions reflecting the workers’ average daily time use were constructed:

1. LIPA 1 (ie, count‐based), MVPA, SB, and time in bed (4‐part composition);
2. LIPA 2 (ie, activity type–based incl. moving and slow walking), standing, MVPA, SB, and time in bed (5‐part composition); and
3. LIPA 3 (ie, activity type–based incl. moving, walking slow, and standing), MVPA, SB, and time in bed (4‐part composition).

For composition (1), MVPA and SB were defined as time spent with activity CPM of > 2029 and < 100, respectively. For composition (2) and (3), MVPA was defined as time spent walking fast, running, stair climbing, and cycling, and SB was defined as time spent sitting or lying during waking hours. In all cases, time in bed was defined based on the worker's daily diary.

### 2.8 Potential confounders and descriptive variables

The following variables were considered as potential confounders based on previous research^3^, ^26^ and theoretical considerations: sex, age, smoking status, and poor diet habits. Sex and age of the workers were determined from each worker's unique Danish civil registration number. Information on poor dietary habits was obtained by the question: *How often do you eat/drink:* “candy, ice cream, chocolate, soft drinks” and “fast food, pizza, burger, shawarma etc” with four response categories that we dichotomized into two groups (frequent poor dietary habits: every day to 2‐3 times per week and infrequent poor dietary habits: 1‐2 times per week to rarely). Smoking behavior was determined from the question “Do you smoke?” with four response categories dichotomized into two groups: smokers (yes daily; yes sometimes) and non‐smokers (used to smoke but not anymore; have never smoked).

### 2.9 Statistical analyses

#### 2.9.1 Descriptive statistics

The characteristics of the study population were described using mean with standard deviations (SD) and numbers with proportions (%) where appropriate. Geometric means were calculated for the three daily time‐use compositions to measure the central tendency of the data.^27^, ^28^ They were obtained by computing the geometric mean of each individual part of the respective compositions and then normalizing (closing) these to be expressed in units relative to the workers’ daily time use (ie, 1440 minutes).

### 2.10 Isometric log‐ratio (ilr) transformation and compositional regression

The exposure compositions were transformed using isometric log‐ratio transformation and presented using pivot ilr‐coordinates. This way, the first ilr‐coordinate expresses the first part of the composition relative to the geometric mean of the remaining parts.^29^ In the three compositions, LIPA was placed as the first part and thus the relative importance of LIPA with respect to the remaining parts was represented in the first ilr‐coordinate for subsequent statistical significance testing through regression analyses.

The strength and direction of the associations between LIPA (relative to the remaining activities and behaviors) and adiposity markers were estimated using unadjusted and adjusted compositional linear regression models. Age, sex, smoking status (reference = non‐smoker), and intake of candy and fast food (reference = infrequent) were included as potential confounders in the adjusted models. Missing data were not imputed, and thus, participants with missing data in any of the variables used in the models were excluded from the adjusted models. Beta coefficients, 95% confidence intervals (CI), and 2‐sided Wald test *P*‐values were estimated for the first ilr‐coordinate for all models. The assumptions of normality and homoscedasticity of the residuals were assessed and satisfied for all models by visual inspection of plots of residuals versus predicted values and quantile‐quantile plots (see Figure S1).

### 2.11 Estimated strength of the associations

Based on the adjusted compositional regression models, we estimated the potential difference in adiposity markers when increasing and decreasing time spent on LIPA from its mean value by a fixed duration of time and accordingly increasing/decreasing time spent in either MVPA or SB. A detailed description of the method can be found in Dumuid et al.^30^, ^31^ In short, fixed durations of time expressed in proportions (ie, minutes/1440) were reallocated between SB and LIPA and MVPA and LIPA, respectively, while keeping the proportion of time spent in all other behaviors constant. This way, the total of 1440 minutes was maintained. To ensure that the reallocation was within the range of available data, we reallocated up to 30 minutes between MVPA and LIPA and 90 minutes between SB and LIPA.

The estimated difference in BMI, body fat percentage, and waist circumference associated with reallocated compositions was plotted to display the relationship between the three LIPA definitions and the outcomes. All analyses were performed in R version 3.4.0,^32^ using the compositions^33^ and robCompositions^34^ packages.

## 3 RESULTS

### 3.1 Population characteristics

Mean age of the study population was 45.2 (SD = 9.6) years, 41% were women, 56% had infrequent poor dietary habits, 88% rarely ate fast food, and 33% were smokers. Mean BMI was 27.0 (SD = 4.9) kg/m^2^, mean body fat percentage was 27.0 (SD = 10.2), and mean waist circumference was 95.1 (SD = 13.2) cm (Table 1).

**TABLE 1:** Baseline characteristics of the study population

Variables	N	%	Mean (SD)
Age in years	513	100	45.2 (9.6)
BMI in kg/m^2^	510	99	27.0 (4.9)
Body fat percentage	361	70	27.0 (10.2)
Waist circumference in cm	482	94	95.1 (13.2)
Sex			
Women	210	41	
Men	303	59	
Missing	0	0	
Collar			
Blue‐collar	471	91	
White‐collar	42	9	
Missing	0	0	
Smoking status			
Smoker	163	32	
Non‐smoker	338	66	
Missing	12	2	
Eat/drink candy, ice cream, chocolate, soft drinks			
Regularly	218	42	
Rarely	290	56	
Missing	5	2	
Eat fast food, pizza, burger, shawarma etc			
Regularly	53	10	
Rarely	454	88	
Missing	6	2	
Working sector			
Cleaning	100	19	
Manufacturing	176	34	
Transportation	81	16	
Health Service	17	3	
Assemblers	33	7	
Construction	41	8	
Garbage Collectors	29	6	
Mobile Plant Operators	11	2	
Other^a^	25	5	
Missing	0	0	

Abbreviations: %, percentage of study sample; N, number in study sample; SD, standard deviation.

^a^ Includes general office clerks and other elementary workers.

### 3.2 Compositional descriptive

Compositional means of the three compositions are shown in Table 2. When using a count‐based definition of LIPA (LIPA 1), the workers spent on average 23% of their day on LIPA. In contrast, only 7% of the day was spent on LIPA when defining LIPA as time spent moving or walking slow (LIPA 2).

**TABLE 2:** Geometric means of daily time spent in moderate‐to‐vigorous physical activity (MVPA), sedentary behaviors, and time in bed with each light‐intensity physical activity (LIPA) definition

	Min./day	%
LIPA 1: count‐based		
LIPA	326	23
MVPA^a^	31	2
SB^b^	700	49
Time in bed	383	26
LIPA 2: posture‐based; moving and walking slow		
LIPA	102	7
Standing	168	12
MVPA^c^	89	6
SB^d^	694	48
Time in bed	387	27
LIPA 3: posture‐based; moving, walking slow, and standing		
LIPA	274	19
MVPA^c^	89	6
SB^d^	692	48
Time in bed	386	27

Abbreviation: LIPA, light‐intensity physical activity.

^a^ MVPA = count‐based using cut‐point of > 2029 CPM.

^b^ SB = count‐based using cut‐point of < 100 CPM.

^c^ MVPA = walking fast, running, stair climbing, and cycling.

^d^ SB = sedentary behavior (sitting and lying).

### 3.3 Compositional linear regression

Results of unadjusted and adjusted compositional linear models with each LIPA definitions are shown in Table 3. The directions and effect estimates of the associations between LIPA and adiposity markers differed depending on how LIPA was defined. The relative importance of count‐based LIPA 1 in the daily time‐use composition was associated with higher BMI (beta: 1.59, 95% CI: 0.04; 3.14), higher body fat percentage (beta: 2.58, 95% CI: −0.14; 5.29), and larger waist circumference (beta: 3.10, 95% CI: −1.14; 7.34). Moreover, the largest effect sizes were found when assessing the association between the relative importance of LIPA 1 and BMI body fat percentage and waist circumference. In contrast, smaller effect sizes of opposite direction were found for the association between the relative importance of daily time spent on LIPA and BMI, body fat percentage, and waist circumference when using the activity type–based LIPA 2 and LIPA 3 (Table 3).

**TABLE 3:** Compositional regression analysis: adjusted and unadjusted associations between the relative importance of the three light‐intensity physical activity (LIPA) definitions and adiposity markers

Adiposity marker	Model	N	LIPA 1			LIPA 2			LIPA 3		
			$\hat{\beta }$	95% CI	*P*‐value	$\hat{\beta }$	95% CI	*P*‐value	$\hat{\beta }$	95% CI	*P*‐value
BMI	Unadjusted	510	1.38	[−0.14; 2.89]	.08	−1.68	[−3.20; −0.17]	.03	−1.36	[−2.88; 0.16]	.08
	Adjusted	492	1.59	[0.04; 3.14]	.04*	−1.29	[−2.78; 0.21]	.09	−1.34	[−2.88; 0.20]	.09
Body fat percentage	Unadjusted	361	6.16	[2.79; 9.54]	<.01**	−6.10	[−9.79; −2.40]	<.01*	1.89	[−1.84; 5.57]	.32
	Adjusted	345	2.58	[−0.14; 5.29]	.06	−1.97	[−4.72; 0.78]	.16	−0.42	[−3.13; 2.30]	.76
Waist circumference	Unadjusted	482	0.44	[−3.96; 4.83]	.85	−0.17	[−4.34; 4.00]	.94	−3.12	[−7.23; 0.98]	.14
	Adjusted	464	3.10	[−1.14; 7.34]	.15	−0.26	[−4.21; 3.69]	.90	−0.70	[−4.70; 3.29]	.73

Abbreviations: BMI, body mass index; LIPA, light‐intensity physical activity; SE, standard error.

LIPA 1 = count‐based using cut‐point of 100‐2029 CPM. LIPA 2 = moving and walking slow. LIPA 3 = moving, walking slow, and standing. $\hat{\beta }$ = beta‐coefficient of the ilr_1_‐coordinate of the daily time‐use composition. Waist circumference is in centimeters. N = number of observations included in the model. Regression models adjusted for age, sex, smoking status, and diet.

* *P* < .05.

** *P* < .01.

### 3.4 Time reallocations

**FIGURE 2:**
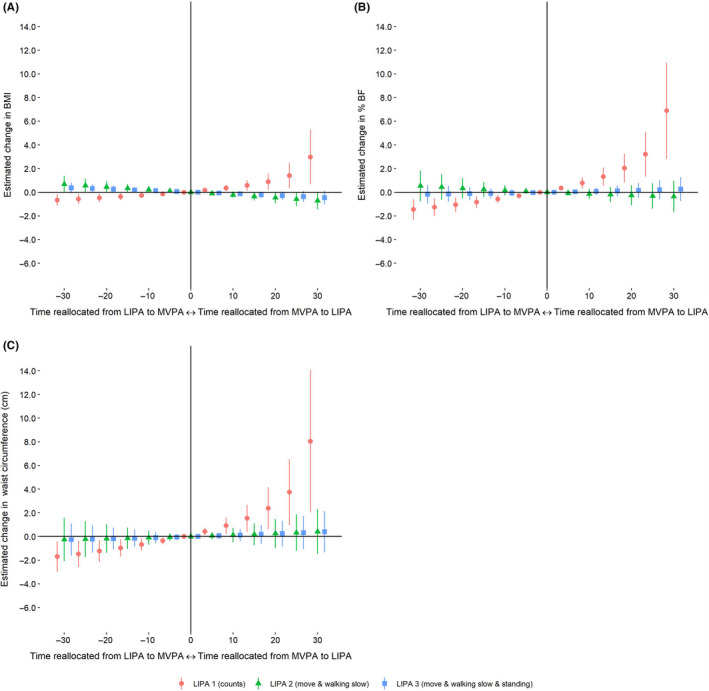
Estimated change in (A) BMI, (B) body fat percentage (% BF), and (C) waist circumference when reallocating time between moderate‐to‐vigorous physical activity (MVPA) and light physical activity (LIPA)

**FIGURE 3:**
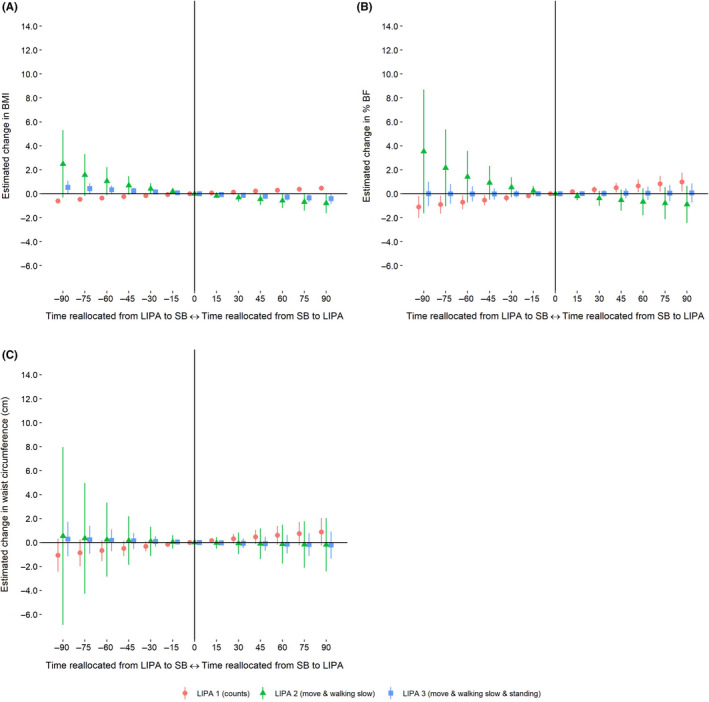
Estimated change in (A) BMI, (B) body fat percentage (% BF), and (C) waist circumference when reallocating time between sedentary behavior (SB) and light physical activity (LIPA)

Reallocating time from MVPA to LIPA 1 was associated with a higher BMI, body fat percentage, and waist circumference, respectively (Figure 2). In contrast, reallocating time from MVPA to LIPA 2, and to LIPA 3 was associated with a lower BMI but showed no associations with body fat percentage nor waist circumference (Figure 2B,C). Reallocating time from SB to LIPA 1 was associated with a higher BMI and body fat percentage (Figure 3A,B), whereas reallocating time from SB to the activity type–based LIPA definitions were associated with a lower BMI (Figure 3A). Estimated differences in BMI, body fat percentage, and waist circumference associated with each time reallocations for all LIPA definitions are presented in an additional file (see Table S2).

## 4 DISCUSSION

In this study, we found that the association between relative daily time spent in LIPA and adiposity markers depended on the LIPA definition. Reallocating time from MVPA to LIPA was associated with increases in all adiposity markers when using the count‐based LIPA, whereas a decrease in BMI was observed when LIPA was based on activity types. Moreover, reallocating time from SB to the count‐based LIPA was associated with increases in BMI and body fat percentage, while reallocating time from SB to LIPA based on activity types was associated with a decrease in BMI.

A potential reason for our finding is the discrepancies in estimated daily time use arising from the different LIPA definitions implemented. We observed considerably different LIPA estimates provided by each definition. For example, based on the count‐based definition, an average of 326 min/day was spent in LIPA. In contrast, based on definition of time spent on moving or walking slow, only 102 min/day was spent in LIPA on average. Moreover, estimated daily time spent in MVPA also differed considerably between the compositions, ranging from 89 min/day (activity type–based) to 31 min/day (count‐based). MVPA has been shown to have significant positive effects on adiposity markers, such as waist circumference,^35^ body fat percentage,^35^ and abdominal visceral fat^36^, ^37^ and is likely to have a more potent health effect per time unit compared with LIPA.^38^, ^39^ In line with this, we found stronger associations between LIPA and adiposity markers when reallocating time from MVPA to LIPA compared to reallocating time from SB to LIPA. Thus, the observed differences in associations between daily time spent in each LIPA definition and adiposity markers could be attributed to discrepancies in time available for the remaining behaviors.

The finding of a higher BMI and body fat percentage associated with reallocating time from SB to count‐based LIPA is counterintuitive. Nevertheless, these results suggest that the count‐based definition of LIPA is more closely associated with the tested adiposity markers compared to the activity type–based definitions of LIPA. Adiposity markers are commonly known to be associated with energy expenditure, to which count‐based methods are also closely linked. However, since counts are only linearly correlated with energy expenditure within a specific activity type, a more accurate representation of LIPA may be achieved through a combination of count and activity/body posture information.^40^

### 4.1 Strength and limitations

A major strength of our study was the use of hip‐ and thigh‐worn accelerometers, enabling assessment of daily time spent in LIPA based on either counts or activity types. Using compositional data analysis facilitated assessment of the association between relative daily time spent in LIPA and adiposity markers, taking other daily physical behaviors (ie, MVPA, SB, and time in bed) into account.

The main limitation of this study was the lack of a “gold standard” LIPA definition. Consequently, we are unable to state which LIPA definition to be the most reliable. Moreover, our study population consisted of mainly blue‐collar workers, thereby limiting comparability with previous studies which are predominantly based on white‐collar workers. The cross‐sectional design of the study could be considered a limitation as it hinders inference of causality. Nevertheless, the study design should not influence the findings of our study.

## 5 PERSPECTIVE

Our findings of conflicting associations between differing LIPA definitions and adiposity markers are in line with those from previous reviews on the health effects of objectively measured LIPA.^3^, ^4^ For example, one review found inconsistent associations between LIPA and BMI due to discrepancies in how LIPA was defined and analyzed between studies.^3^ Another review consistently found negative associations between LIPA and waist circumference and BMI.^4^ However, this review only included studies based on accelerometer data from NHANES and count‐based measurements of LIPA, thereby minimizing methodological discrepancies between the studies. Clearly, current evidence on the effect of LIPA on adiposity markers is ambiguous. Developing standardized methods for analyzing objective measurements of LIPA could decrease this ambiguity through facilitating comparability and synthesis of results. Furthermore, a combination of count‐ and activity type–based measurements could give a more accurate presentation of LIPA.

## CONFLICT OF INTEREST

The authors declare that they have no conflicts of interest.

## Supporting information

Table S1

Table S2

Fig S1
