# Supplementary material for: Light‐intensity physical activity derived from count or activity types is differently associated with adiposity markers
Source: Scand J Med Sci Sports. 2020 Jul 5;30(10):1966–75. doi: 10.1111/sms.13743 (PMC7540429; doi:10.1111/sms.13743)
Supplement: Supplementary file 1 — Table S1 [file SMS-30-1966-s001.docx]

**Additional file 1**

Comparison of individuals included and excluded in the study based on the criterion of having both hip- and thigh-worn accelerometer data.

**Table S1.** Comparison on individuals with (i.e. included in the study, n=513) and without hip- and thigh-worn accelerometers (i.e. excluded from the study, n=619).

|  | **Included (513)** | | **Excluded (n=619)** | |
| --- | --- | --- | --- | --- |
| **Variables** | **N (%)** | **Mean (SD)** | **N (%)** | **Mean (SD)** |
| Age in years | 513 (100) | 45.2 (9.6) | 619 (100) | 44.7 (10.2) |
| BMI in kg/m^2^ | 510 (99) | 27.0 (4.9) | 604 (98) | 27.2 (4.8) |
| Body fat percentage (%) | 361 (70) | 27.0 (10.2) | 472 (76) | 30.4 (9.0) |
| Waist circumcise (cm) | 482 (94) | 95.1 (13.2) | 364 (59) | 92.4 (12.8) |
| Sex |  |  |  |  |
| Women | 210 (41) |  | 318 (51) |  |
| Men | 303 (59) |  | 285 (46) |  |
| Missing | 0 (0) |  | 16 (3) |  |
| Collar |  |  |  |  |
| Blue-collar | 471 (91) |  | 479 (77) |  |
| White-collar | 42 (9) |  | 124 (33) |  |
| Missing | 0 (0) |  | 0 (0) |  |
| Smoking status |  |  |  |  |
| Smoker | 163 (32) |  | 181 (29) |  |
| Non-smoker | 338 (66) |  | 436 (70) |  |
| Missing | 12 (2) |  | 2 (1) |  |
| Eat/drink candy, ice cream, chocolate, soft drinks |  |  |  |  |
| Regularly | 218 (42) |  | 238 (38) |  |
| Rarely | 290 (56) |  | 364 (59) |  |
| Missing | 5 (2) |  | 17 (3) |  |
| Eat fast food, pizza, burger, shawarma etc. |  |  |  |  |
| Regularly | 53 (10) |  | 39 (6) |  |
| Rarely | 454 (88) |  | 563 (91) |  |
| Missing | 6 (2) |  | 17 (3) |  |
