# Supplementary material for: Light‐intensity physical activity derived from count or activity types is differently associated with adiposity markers
Source: Scand J Med Sci Sports. 2020 Jul 5;30(10):1966–75. doi: 10.1111/sms.13743 (PMC7540429; doi:10.1111/sms.13743)
Supplement: Supplementary file 2 — Table S2 [file SMS-30-1966-s002.docx]

**Additional file 3**

**Table S1.** Estimated change in adiposity indicators when reallocating time between moderate-to-vigorous physical activity (MVPA) and each definition of light physical activity (LIPA).

| Time reallocation between MVPA and LIPA (min) | Estimated BMI change | 95 % CI | Estimated body fat % change | 95 % CI | Estimated waist cm change | 95 % CI |  |
| --- | --- | --- | --- | --- | --- | --- | --- |
| *LIPA 1: count-based* | | | | | | | |
| -30 (MVPA🡨LIPA) | -0.66 | (-1.14;-0.18) | -1.45 | (-2.31;-0.60) | -1.70 | (-2.98;-0.43) |  |
| -25 (MVPA🡨LIPA) | -0.57 | (-0.10;-0.15) | -1.26 | (-2.00;-0.52) | -1.47 | (-2.58;-0.37) |  |
| -20 (MVPA🡨LIPA) | -0.48 | (-0.82;-0.13) | -1.05 | (-1.67;-0.43) | -1.23 | (-2.15;-0.31) |  |
| -15 (MVPA🡨LIPA) | -0.37 | (-0.65;-0.10) | -0.83 | (-1.31;-0.34) | -0.97 | (-1.7;-0.25) |  |
| -10 (MVPA🡨LIPA) | -0.26 | (-0.45;-0.07) | -0.58 | (-0.92;-0.24) | -0.68 | (-1.19;-0.17) |  |
| -5 (MVPA🡨LIPA) | -0.14 | (-0.24;-0.04) | -0.31 | (-0.49;-0.13) | -0.36 | (-0.63;-0.09) |  |
| 0 | - | - | - | - | - | - |  |
| 5 (MVPA🡪LIPA) | 0.16 | (0.04;0.28) | 0.36 | (0.15;0.57) | 0.42 | (0.11;0.73) |  |
| 10 (MVPA🡪LIPA) | 0.35 | (0.09;0.61) | 0.78 | (0.32;1.24) | 0.91 | (0.23;1.59) |  |
| 15 (MVPA🡪LIPA) | 0.58 | (0.14;1.02) | 1.32 | (0.54;2.09) | 1.54 | (0.39;2.70) |  |
| 20 (MVPA🡪LIPA) | 0.90 | (0.22;1.57) | 2.04 | (0.84;3.24) | 2.38 | (0.61;4.16) |  |
| 25 (MVPA🡪LIPA) | 1.40 | (0.33;2.46) | 3.21 | (1.32;5.09) | 3.75 | (0.96;6.53) |  |
| 30 (MVPA🡪LIPA) | 2.97 | (0.68;5.27) | 6.89 | (2.82;10.96) | 8.04 | (2.04;14.04) |  |
| *LIPA 2: posture based; moving and walking slow* | | | | | | | |
| -30 (MVPA🡨LIPA) | 0.69 | (-0.01;1.39) | 0.54 | (-0.74;1.83) | -0.25 | (-2.08;1.58) |  |
| -25 (MVPA🡨LIPA) | 0.57 | (-0.01;1.15) | 0.44 | (-0.63;1.50) | -0.22 | (-1.73;1.29) |  |
| -20 (MVPA🡨LIPA) | 0.45 | (-0.01;0.91) | 0.34 | (-0.51;1.18) | -0.18 | (-1.38;1.02) |  |
| -15 (MVPA🡨LIPA) | 0.34 | (-0.01;0.68) | 0.24 | (-0.39;0.87) | -0.14 | (-1.04;0.75) |  |
| -10 (MVPA🡨LIPA) | 0.23 | (-0.004;0.45) | 0.16 | (-0.26;0.57) | -0.10 | (-0.69;0.50) |  |
| -5 (MVPA🡨LIPA) | 0.11 | (-0.002;0.23) | 0.08 | (-0.13;0.28) | -0.05 | (-0.35;0.25) |  |
| 0 | - | - | - | - | - | - |  |
| 5 (MVPA🡪LIPA) | -0.11 | (-0.23;0.002) | -0.07 | (-0.28;0.14) | 0.06 | (-0.24;0.35) |  |
| 10 (MVPA🡪LIPA) | -0.23 | (-0.46;0.01) | -0.14 | (-0.56;0.29) | 0.12 | (-0.48;0.71) |  |
| 15 (MVPA🡪LIPA) | -0.34 | (-0.69;0.01) | -0.20 | (-0.83;0.44) | 0.18 | (-0.73;1.09) |  |
| 20 (MVPA🡪LIPA) | -0.46 | (-0.93;0.01) | -0.26 | (-1.11;0.60) | 0.25 | (-0.97;1.47) |  |
| 25 (MVPA🡪LIPA) | -0.58 | (-1.175;0.01) | -0.31 | (-1.39;0.78) | 0.33 | (-1.22;1.87) |  |
| 30 (MVPA🡪LIPA) | -0.71 | (-1.43;0.02) | -0.36 | (-1.68;0.96) | 0.41 | (-1.47;2.29) |  |
| *LIPA 3: posture based; moving, walking slow and standing* | | | | | | | |
| -30 (MVPA🡨LIPA) | 0.37 | (-0.07;0.81) | -0.17 | (-0.95;0.62) | -0.26 | (-1.62;1.10) |  |
| -25 (MVPA🡨LIPA) | 0.31 | (-0.06;0.68) | -0.14 | (-0.81;0.52) | -0.22 | (-1.37;0.93) |  |
| -20 (MVPA🡨LIPA) | 0.25 | (-0.05;0.55) | -0.12 | (-0.66;0.42) | -0.18 | (-1.12;0.75) |  |
| -15 (MVPA🡨LIPA) | 0.19 | (-0.04;0.42) | -0.09 | (-0.50;0.32) | -0.14 | (-0.85;0.57) |  |
| -10 (MVPA🡨LIPA) | 0.13 | (-0.03;0.29) | -0.06 | (-0.34;0.21) | -0.10 | (-0.58;0.38) |  |
| -5 (MVPA🡨LIPA) | 0.07 | (-0.01;0.15) | -0.03 | (-0.17;0.11) | -0.05 | (-0.30;0.19) |  |
| 0 | - | - | - | - | - | - |  |
| 5 (MVPA🡪LIPA) | -0.07 | (-0.15;0.02) | 0.04 | (-0.11;0.18) | 0.06 | (-0.20;0.31) |  |
| 10 (MVPA🡪LIPA) | -0.14 | (-0.31;0.03) | 0.07 | (-0.23;0.37) | 0.11 | (-0.41;0.63) |  |
| 15 (MVPA🡪LIPA) | -0.21 | (-0.47;0.05) | 0.11 | (-0.35;0.57) | 0.18 | (-0.62;0.98) |  |
| 20 (MVPA🡪LIPA) | -0.29 | (-0.64;0.07) | 0.16 | (-0.47;0.79) | 0.25 | (-0.85;1.34) |  |
| 25 (MVPA🡪LIPA) | -0.37 | (-0.82;0.09) | 0.21 | (-0.60;1.02) | 0.32 | (-1.08;1.73) |  |
| 30 (MVPA🡪LIPA) | -0.45 | (-1.01;0.11) | 0.26 | (-0.74;1.26) | 0.41 | (-1.33;2.14) |  |

*95% CI = 95% confidence interval for the expected change. (MVPA🡨LIPA) means time was reallocated from LIPA to MVPA. (MVPA🡪LIPA) means time was reallocated from MVPA to LIPA.*

**Table B2.** Estimated change in adiposity indicators when reallocating time between light intensity physical activity (LIPA) and sedentary behaviour (SB).

| Time reallocation between SB and LIPA (min) | Estimated BMI change | 95 % CI | Estimated body fat % change | 95 % CI | Estimated waist cm change | 95 % CI |
| --- | --- | --- | --- | --- | --- | --- |
| *LIPA 1: count-based* | | | | | | |
| -90 (SB🡨LIPA) | -0.61 | (-0.73; -0.48) | -1.10 | (-2.01; -0.19) | -1.07 | (-2.43; 0.30) |
| -75 (SB🡨LIPA) | -0.47 | (-0.56; -0.39) | -0.91 | (-1.65; -0.16) | -0.87 | (-1.98; 0.24) |
| -60 (SB🡨LIPA) | -0.36 | (-0.41; -0.30) | -0.71 | (-1.30; -0.13) | -0.68 | (-1.56; 0.19) |
| -45 (SB🡨LIPA) | -0.25 | (-0.28; -0.22) | -0.53 | (-0.96; -0.02) | -0.50 | (-1.15; 0.14) |
| -30 (SB🡨LIPA) | -0.16 | (-0.17; -0.15) | -0.35 | (-0.63; -0.07) | -0.33 | (-0.75; 0.09) |
| -15 (SB🡨LIPA) | -0.07 | (-0.08; -0.07) | -0.17 | (-0.31; -0.03) | -0.16 | (-0.37; 0.05) |
| 0 | - | - | - | - | - | - |
| 15 (SB🡪 LIPA) | 0.07 | (0.07; 0.07) | 0.17 | (0.03; 0.31) | 0.16 | (-0.05; 0.36) |
| 30 (SB🡪LIPA) | 0.15 | (0.13; 0.16) | 0.34 | (0.07; 0.61) | 0.31 | (-0.09; 0.71) |
| 45 (SB🡪LIPA) | 0.22 | (0.20; 0.25) | 0.50 | (0.02; 0.90) | 0.46 | (-0.13; 1.05) |
| 60 (SB🡪LIPA) | 0.30 | (0.26; 0.34) | 0.66 | (0.13; 1.19) | 0.60 | (-0.18; 1.38) |
| 75 (SB🡪LIPA) | 0.38 | (0.32; 0.44) | 0.82 | (0.16; 1.48) | 0.74 | (-0.22; 1.71) |
| 90 (SB🡪LIPA) | 0.46 | (0.31; 0.55) | 0.98 | (0.20; 1.76) | 0.88 | (-0.27; 2.03) |
| *LIPA 2: posture based; moving and walking slow* | | | | | | |
| -90 (SB🡨LIPA) | 2.49 | (-0.32;5.30) | 3.54 | (-1.62;8.69) | 0.54 | (-6.88;7.95) |
| -75 (SB🡨LIPA) | 1.56 | (-0.18;3.31) | 2.16 | (-1.04;5.37) | 0.34 | (-4.28;4.96) |
| -60 (SB🡨LIPA) | 1.05 | (-0.12;2.22) | 1.42 | (-0.72;3.56) | 0.23 | (-2.85;3.32) |
| -45 (SB🡨LIPA) | 0.69 | (-0.07;1.46) | 0.92 | (-0.48;2.32) | 0.16 | (-1.87;2.18) |
| -30 (SB🡨LIPA) | 0.42 | (-0.04;0.88) | 0.54 | (-0.30;1.38) | 0.10 | (-1.12;1.31) |
| -15 (SB🡨LIPA) | 0.19 | (-0.02;0.40) | 0.25 | (-0.140;0.63) | 0.04 | (-0.51;0.56) |
| 0 | - | - | - | - | - | - |
| 15 (SB🡪 LIPA) | -0.17 | (-0.35;0.01) | -0.21 | (-0.54;0.13) | -0.04 | (-0.52;0.44) |
| 30 (SB🡪LIPA) | -0.32 | (-0.66;0.03) | -0.38 | (-1.01;0.24) | -0.07 | (-0.98;0.83) |
| 45 (SB🡪LIPA) | -0.45 | (-0.93;0.04) | -0.53 | (-1.42;0.35) | -0.11 | (-1.38;1.17) |
| 60 (SB🡪LIPA) | -0.57 | (-1.18;0.04) | -0.67 | (-1.79;0.46) | -0.14 | (-1.76;1.48) |
| 75 (SB🡪LIPA) | -0.68 | (-1.41;0.05) | -0.78 | (-2.12;0.56) | -0.17 | (-2.10;1.77) |
| 90 (SB🡪LIPA) | -0.79 | (-1.63;0.06) | -0.89 | (-2.42;0.65) | -0.19 | (-2.42;2.03) |
| *LIPA 3: posture based; moving, walking slow and standing* | | | | | | |
| -90 (SB🡨LIPA) | 0.53 | (-0.03;1.08) | -0.01 | (-1.01;0.10) | 0.28 | (-1.17;1.73) |
| -75 (SB🡨LIPA) | 0.43 | (-0.02;0.88) | -0.01 | (-0.83;0.80) | 0.23 | (-0.94;1.39) |
| -60 (SB🡨LIPA) | 0.33 | (-0.01;0.68) | -0.01 | (-0.65;0.62) | 0.18 | (-0.73;1.08) |
| -45 (SB🡨LIPA) | 0.24 | (-0.01;0.50) | -0.01 | (-0.48;0.45) | 0.13 | (-0.53;0.79) |
| -30 (SB🡨LIPA) | 0.16 | (-0.01;0.32) | -0.01 | (-0.31;0.29) | 0.08 | (-0.35;0.52) |
| -15 (SB🡨LIPA) | 0.08 | (-0.004;0.16) | -0.01 | (-0.15;0.14) | 0.04 | (-0.17;0.25) |
| 0 | - | - | - | - | - | - |
| 15 (SB🡪 LIPA) | -0.07 | (-0.15;0.004) | 0.01 | (-0.13;0.15) | -0.04 | (-0.24;0.16) |
| 30 (SB🡪LIPA) | -0.15 | (-0.30;0.01) | 0.02 | (-0.26;0.30) | -0.08 | (-0.47;0.32) |
| 45 (SB🡪LIPA) | -0.21 | (-0.44;0.01) | 0.03 | (-0.38;0.44) | -0.11 | (-0.70;0.47) |
| 60 (SB🡪LIPA) | -0.28 | (-0.57;0.02) | 0.04 | (-0.50;0.58) | -0.15 | (-0.92;0.62) |
| 75 (SB🡪LIPA) | -0.34 | (-0.71;0.02) | 0.05 | (-0.61;0.72) | -0.18 | (-1.13;0.77) |
| 90 (SB🡪LIPA) | -0.41 | (-0.84;0.02) | 0.07 | (-0.72;0.86) | -0.22 | (-1.34;0.91) |

*95% CI = 95% confidence interval for the expected change. (SB🡨LIPA) means time was reallocated from LIPA to SB. (SB🡪LIPA) means time was reallocated from SB to LIPA.*
