# Supplementary material for: Light‐intensity physical activity derived from count or activity types is differently associated with adiposity markers
Source: Scand J Med Sci Sports. 2020 Jul 5;30(10):1966–75. doi: 10.1111/sms.13743 (PMC7540429; doi:10.1111/sms.13743)

**Additional file 2**

The following show the diagnostic plots of the models used in the current study. The plots visualize whether assumptions of normality and homoscedasticity of the residuals were satisfied by plotting residuals versus predicted values and quantile-quantile values.

**BMI as outcome**

*Model 1a, LIPA 1 (i.e. count-based) as exposure, adjusted for age, sex, smoking status and diet.*


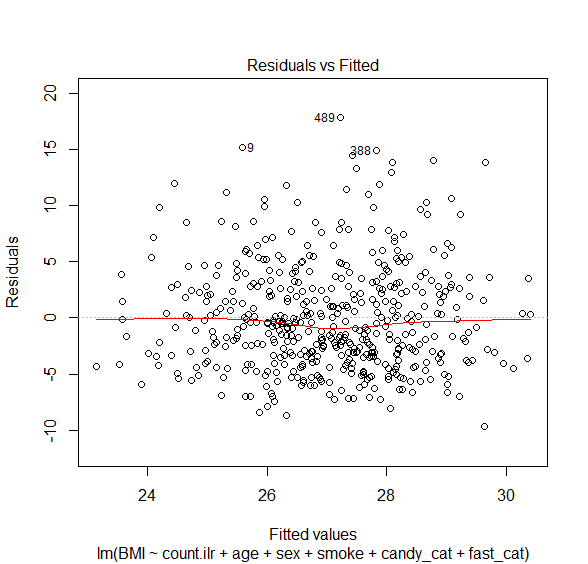

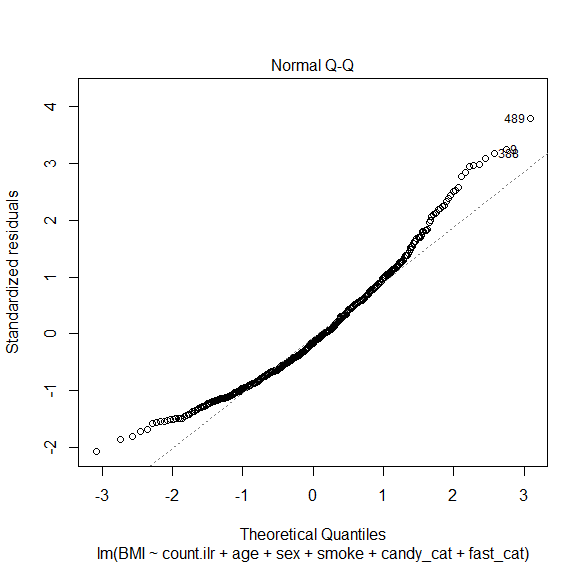


*Model 1b, LIPA 2 (i.e. activity-based; walking slow and moving) as exposure, adjusted for age, sex, smoking status and diet.*


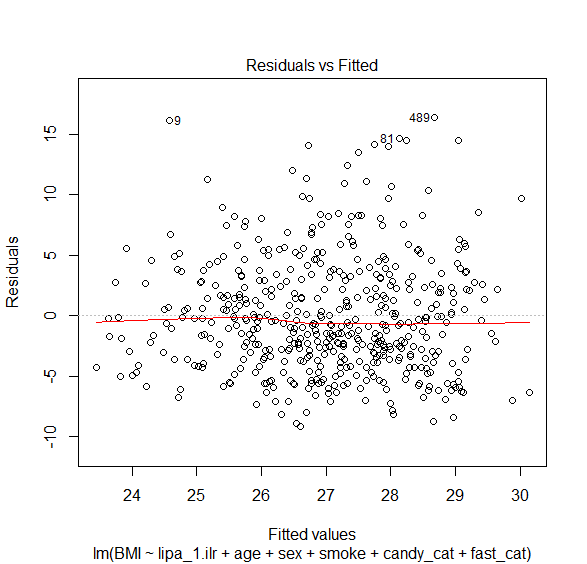

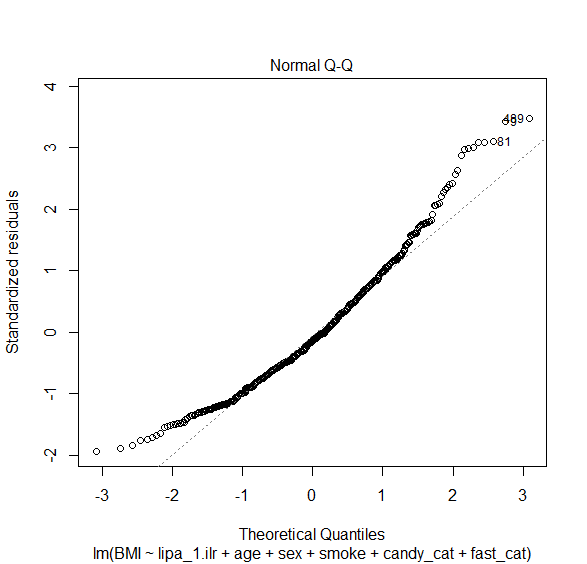


*Model 1c, LIPA 3 (i.e. activity-based; walking slow, moving and standing) as exposure, adjusted for age, sex, smoking status and diet.*


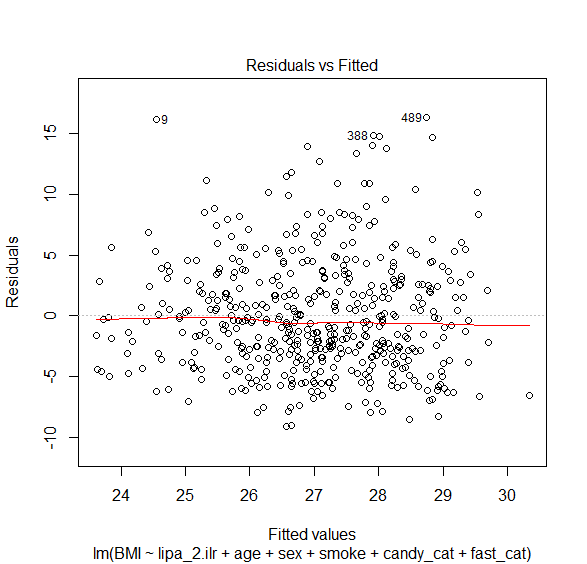

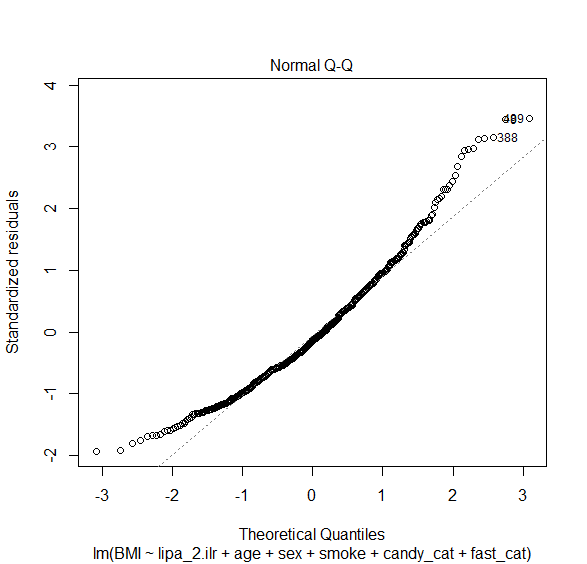


**Body fat percentage as outcome**

*Model 2a, LIPA 1 (i.e. count-based) as exposure, adjusted for age, sex, smoking status and diet.*


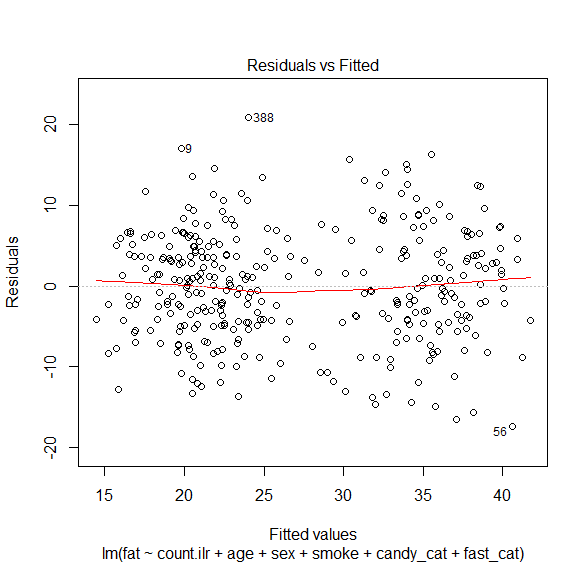

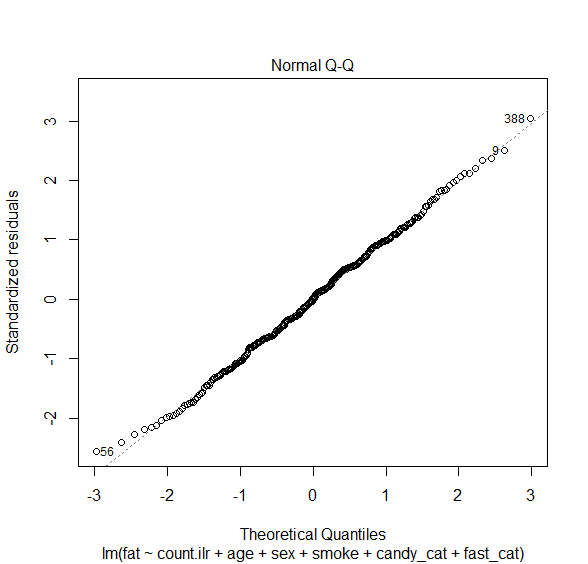


*Model 2b, LIPA 2 (i.e. activity-based; walking slow and moving) as exposure, adjusted for age, sex, smoking status and diet.*


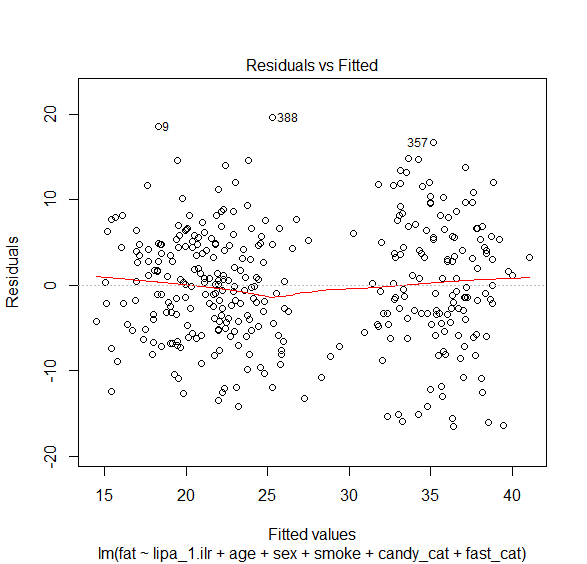

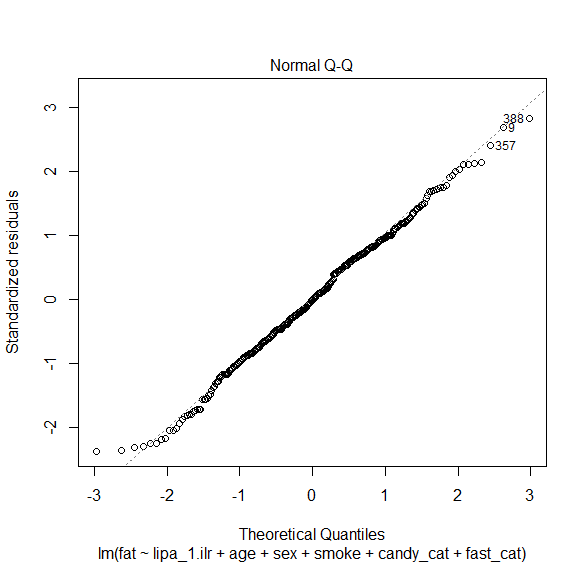


*Model 2c, LIPA 3 (i.e. activity-based; walking slow, moving and standing) as exposure, adjusted for age, sex, smoking status and diet.*


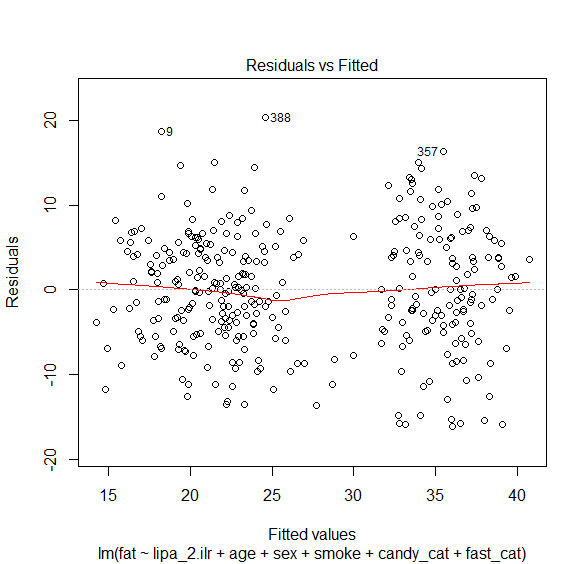

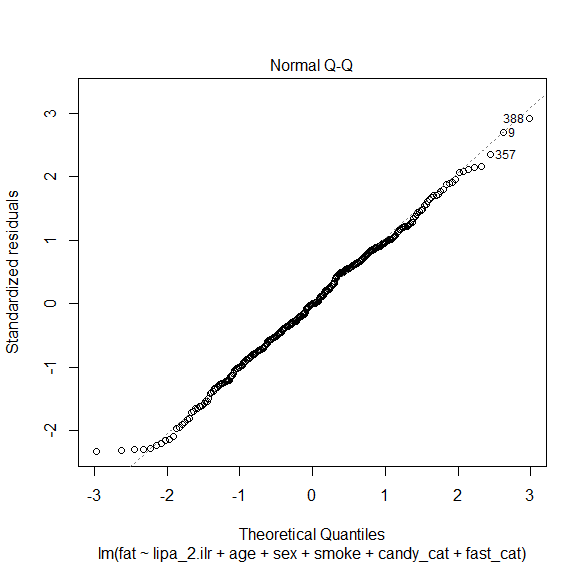


**Waist circumference as outcome**

*Model 3a, LIPA 1 (i.e. count-based) as exposure, adjusted for age, sex, smoking status and diet.*


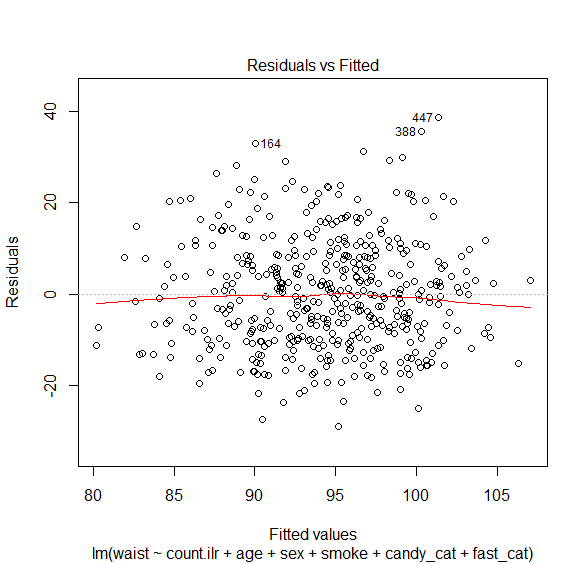

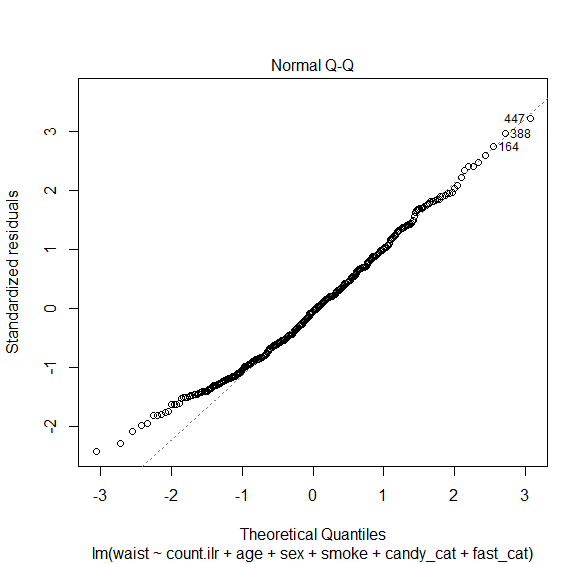


*Model 3b, LIPA 2 (i.e. activity-based; walking slow and moving) as exposure, adjusted for age, sex, smoking status and diet.*


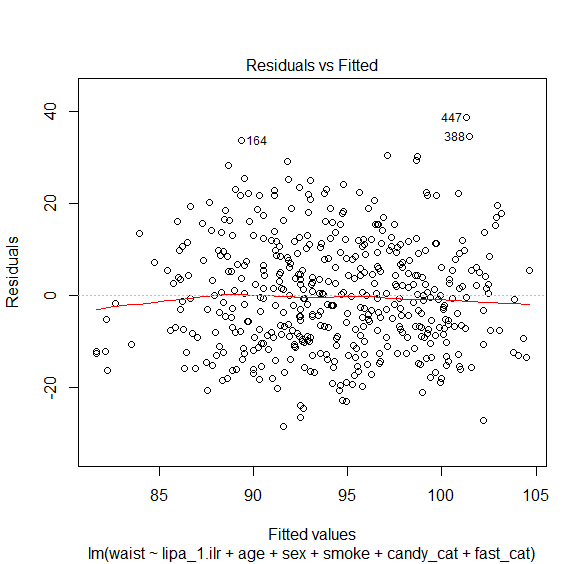

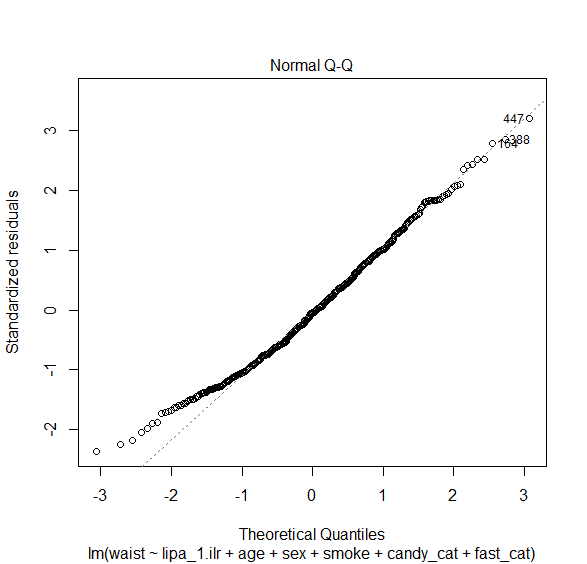


*Model 3c, LIPA 3 (i.e. activity-based; walking slow, moving and standing) as exposure, adjusted for age, sex, smoking status and diet.*


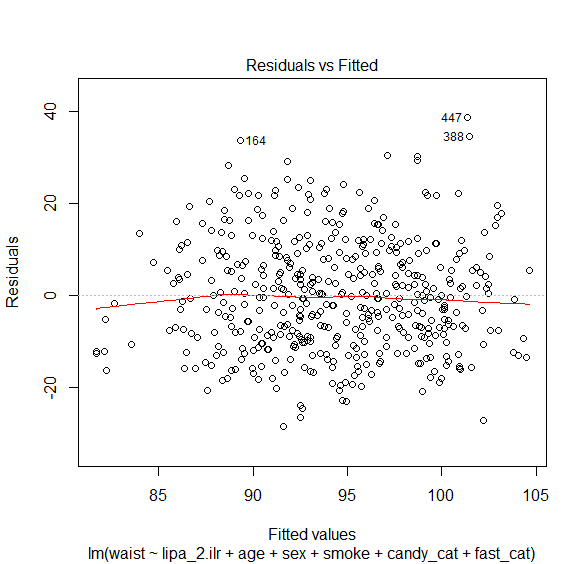

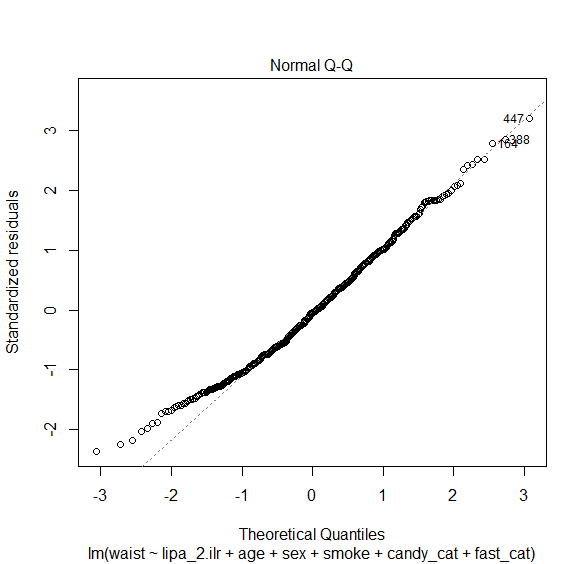

Supplement: Supplementary file 3 — Fig S1 [file SMS-30-1966-s003.docx]
